# Intra-articular injection of two different doses of autologous bone marrow mesenchymal stem cells versus hyaluronic acid in the treatment of knee osteoarthritis: multicenter randomized controlled clinical trial (phase I/II)

**DOI:** 10.1186/s12967-016-0998-2

**Published:** 2016-08-26

**Authors:** José M. Lamo-Espinosa, Gonzalo Mora, Juan F. Blanco, Froilán Granero-Moltó, Jorge M. Nuñez-Córdoba, Carmen Sánchez-Echenique, José M. Bondía, Jesús Dámaso Aquerreta, Enrique J. Andreu, Enrique Ornilla, Eva M. Villarón, Andrés Valentí-Azcárate, Fermín Sánchez-Guijo, María Consuelo del Cañizo, Juan Ramón Valentí-Nin, Felipe Prósper

**Affiliations:** 1 Department of Orthopaedic Surgery and Traumatology, Clínica Universidad de Navarra, Pamplona, Spain; 2 Department of Orthopaedic Surgery and Traumatology, IBSAL-Hospital Universitario de Salamanca, Salamanca, Spain; 3 TerCel (Spanish Cell Therapy Network, Spanish National Institute of Health Carlos III), Madrid, Spain; 4 Cell Therapy Area, Clínica Universidad de Navarra, Pamplona, Spain; 5 Navarra Institute for Health Research (IdiSNA), Pamplona, Spain; 6 Division of Biostatistics, Research Support Service, Central Clinical Trials Unit, Clínica Universidad de Navarra, Pamplona, Spain; 7 Department of Preventive Medicine and Public Health, Medical School, University of Navarra, Pamplona, Spain; 8 Department of Radiology, Clínica Universidad de Navarra, Pamplona, Spain; 9 Department of Rheumatology, Clínica Universidad de Navarra, Pamplona, Spain; 10 Department of Hematology, IBSAL-Hospital Universitario de Salamanca, Salamanca, Spain; 11 Department of Hematology, Clínica Universidad de Navarra, Avenida Pío XII 36, 31009 Pamplona, Navarra Spain; 12 Centro en Red de Medicina Regenerativa y Terapia Celular de Castilla y León, Castilla y León, Salamanca, Spain

**Keywords:** Bone marrow-mesenchymal stromal cells, Knee osteoarthritis, Non-surgical management, Stem cell therapy

## Abstract

**Background:**

Mesenchymal stromal cells are a promising option to treat knee osteoarthritis. Their safety and usefulness must be confirmed and the optimal dose established. We tested increasing doses of bone marrow mesenchymal stromal cells (BM-MSCs) in combination with hyaluronic acid in a randomized clinical trial.

**Materials:**

A phase I/II multicenter randomized clinical trial with active control was conducted. Thirty patients diagnosed with knee OA were randomly assigned to intraarticularly administered hyaluronic acid alone (control), or together with 10 × 10^6^ or 100 × 10^6^ cultured autologous BM-MSCs, and followed up for 12 months. Pain and function were assessed using VAS and WOMAC and by measuring the knee motion range. X-ray and magnetic resonance imaging analyses were performed to analyze joint damage.

**Results:**

No adverse effects were reported after BM-MSC administration or during follow-up. BM-MSC-administered patients improved according to VAS during all follow-up evaluations and median value (IQR) for control, low-dose and high-dose groups change from 5 (3, 7), 7 (5, 8) and 6 (4, 8) to 4 (3, 5), 2 (1, 3) and 2 (0,4) respectively at 12 months (low-dose vs control group p = 0.005 and high-dose vs control group p < 0.009). BM-MSC-administered patients were also superior according to WOMAC, although improvement in control and low-dose patients could not be significantly sustained beyond 6 months. On the other hand, the BM-MSC high-dose group exhibited an improvement of 16.5 (12, 19) points at 12 months (p < 0.01). Consistent with WOMAC and VAS values, motion ranges remained unaltered in the control group but improved at 12 months with BM-MSCs. X-ray revealed a reduction of the knee joint space width in the control group that was not seen in BM-MSCs high-dose group. MRI (WORMS protocol) showed that joint damage decreased only in the BM-MSC high-dose group, albeit slightly.

**Conclusions:**

The single intraarticular injection of in vitro expanded autologous BM-MSCs together with HA is a safe and feasible procedure that results in a clinical and functional improvement of knee OA, especially when 100 × 10^6^ cells are administered. These results pave the way for a future phase III clinical trial.

*Clinical Trials.gov identifier* NCT02123368. Nº EudraCT: 2009-017624-72

**Electronic supplementary material:**

The online version of this article (doi:10.1186/s12967-016-0998-2) contains supplementary material, which is available to authorized users.

## Background

Osteoarthritis (OA) is a chronic disease involving progressive degeneration of the articular cartilage and subchondral bone, accompanied by synovitis [1]. Due to its avascular nature and the limited self-renewal capacity of chondrocytes, adult articular cartilage presents limited repair capability [2]. Current treatment options for articular cartilage injury and osteoarthritis are aimed to relieve inflammation and pain, but have no effect on the natural progression of the disease [3]. To date, in severe cases of knee OA, knee replacement is the only therapeutic option [4].

During the last two decades focal cartilage defects have been treated using cell therapy and tissue engineering approaches. In this context, autologous chondrocyte implantation (ACI) or matrix-induced autologous chondrocyte (MACI) implantation techniques have been applied with promising results, although the large non-contained cartilage defects found in OA and its own pathogenesis cannot be treated using ACI or MACI [5–7]. The use of intraarticular injections of mesenchymal stromal cells (MSCs) may represent some advantages over chondrocytes in patients with OA. First, because of their ability for self-renewal, the number of cells that can be obtained is increased without cartilage donor site morbidity and with reduced cost [6–8]. Second, MSCs are responsible for the normal turnover and maintenance of adult mesenchymal tissues, including cartilage, and has been suggested that the number of MSCs present in the subchondral bone decreases with age and OA grade, suggesting that such MSCs deficit could prime the degenerative process [9–12]. It has also been proposed that during tissue injury MSCs migrate to participate in the reparative process, giving MSCs a potential therapeutic value when added exogenously [13, 14]. Additionally, cultured MSCs induce in vitro chondrocyte proliferation and extracellular matrix protein synthesis, including aggrecan and type II collagen, which support their critical role in cartilage tissue repair [15, 16].

There is an increasing number of reports on the treatment of OA using MSC, but these are methodologically heterogeneous in dose, cell source, coadjuvants and cell processing methods, which makes it difficult to compare the different studies [17]. In many cases, treatments consist of the administration of bone marrow concentrates as a source of MSCs. However, it is well known that only 0.001 % of the mononuclear cells found in the bone marrow could be considered as MSCs as defined by the ICRS in 2006. Therefore, their number in a bone marrow concentrate is very limited compared to that obtained upon culturing MSCs [18–20]. Only a few studies using MSCs produced by good manufacture practices (GMP) such as advanced cell-therapy products have been reported [21–24]. In addition, there is a need to explore the effect of different cell doses in a randomized way to gain insight into the ideal conditions for knee OA patients to take advantage of MSC therapy. For these reasons, the purpose of this study was to randomly assess the safety, feasibility and efficacy of the intra-articular injection of two different doses of GMP-produced autologous bone marrow MSCs (BM-MSCs) with hyaluronic acid (HA) in patients with knee OA.

## Methods

### Participants and study design

This is a phase I/II randomized clinical trial with active control conducted between August 2012 and October 2014, involving the Clínica Universidad de Navarra (Pamplona, Spain) and IBSAL-Hospital Universitario de Salamanca (Salamanca, Spain). All the procedures were approved by the Institutional Review Board of Navarra and the Spanish Agency of Medicines and Medical Devices (Nº EudraCT: 2009-017624-72, Clinical Trials.gov identifier: NCT02123368). All participants provided written informed consent.

### Criteria for eligibility of patients

Inclusion criteria were as follows: males and females aged 50–80, diagnosis of knee OA according to American College of Rheumatology criteria, visual analogue scale (VAS) joint pain ≥2.5, Kellgren–Lawrence radiological classification scale ≥2, body mass index between 20 and 35 kg/m [2], and availability to be followed during the study period; exclusion criteria were: previous diagnosis of polyarticular disease, severe mechanical extra-articular deformation (>15° *varus*/15° *valgus*), systemic autoimmune rheumatic disease, arthroscopy or intraarticular infiltration in the last 6 months, chronic treatment with immunosuppressive or anticoagulant drugs, corticosteroids treatment in the 3 last months, nonsteroidal anti-inflammatory drugs therapy in the last 15 days, bilateral knee OA requiring treatment in both knees, poorly controlled diabetes mellitus, blood dyscrasias, and allergy to HA or bird proteins.

### Treatment groups

Participants were assigned to comparison groups by an unblinded computer-generated list, based on unrestricted randomization, which was maintained centrally by staff with no clinical involvement in the trial so no center knew the treatment allocation of any patient until the patient had been recruited into the trial.

Three groups were created:

Control group, constituted by patients who received a single intra-articular injection of 60 mg HA (Hyalone^®^) in a final volume of 4 ml.

Low-dose BM-MSCs group, constituted by patients who received a single intra-articular injection of 10 × 10^6^ autologous cultured BM-MSC in 1.5 ml Ringer’s lactate solution, followed by an intraarticular injection of 4 ml HA.

High-dose BM-MSCs group, constituted by patients who received a single intra-articular injection of 100 × 10^6^ autologous cultured BM-MSCs in 3 ml Ringer’s lactate solution, followed by an intraarticular injection of 4 ml HA.

### Sample size calculation

We estimated that a sample size of ten patients per group was required to detect an effect size of 0.6 with a power of 80 %, assuming a balanced allocation to treatment groups, and a 5 % type I error probability.

### Cell culture

BM-MSCs were generated under good manufacturing practice conditions (GMP) with standard operating procedures. Briefly, bone marrow (100 ml) was harvested from the pelvic bone (iliac crest) under sterile conditions. The mononuclear cell fraction was isolated by Ficoll density gradient centrifugation (Ficoll-Paque, GE Healthcare Bio-Sciences AB, Uppsala, Sweden). Cells, ranging between 20 × 10^6^ and 60 × 10^6^, were subsequently seeded in 175 cm^2^ flasks with growth medium, which consisted of αMEM without ribonucleosides (Gibco, Life Technologies, Carlsbad, CA, USA) supplemented with 5 % platelet lisate, 2 units/ml heparin, penicillin–streptomycin at 1 % (Gibco) and 1 ng/ml human fibroblast growth factor (bFGF) (Sigma-Aldrich, St. Louis, MO, USA). The flasks were maintained in culture at 37 °C in 5 % CO_2_ atmosphere. The growth medium was changed every 3–4 days. About 10–15 days later, colonies were formed and the cells were split with TrypLE Select™ (Life Technologies) and seeded at 3000–5000 cells/cm^2^. Once 70–80 % confluence was reached, cells were split again and cultured until they were available at the amounts required to be administered to patients. Finally, cells were harvested with TrypLE Select™, washed three times with PBS and resuspended in Ringer’s lactate buffer (Grifols, Barcelona, Spain) containing 1 % human albumin (Grifols), to be administered within 24 h of harvesting of the cells. Cells were characterized according to ISCT criteria. Cells were then analyzed by flow cytometry (FACSCalibur, BD Biosciences, San José, CA, USA) with the appropriate antibodies (BD Biosciences) to confirm expression of surface markers CD90, CD73 and CD44, as well as absence of CD34 and CD45.

### Cell injection

Cell injection was performed without radiographic guidance through a lateral patellar approach by three different orthopaedic surgeons from both involved centers (Additional file 1: Figure S1), 3–4 weeks after the iliac crest biopsy had been performed. In 90 % of the patients, cells were administered within the first hour after being harvested. For this purpose, a 19 G needle was used in two consecutive intraarticular injections. In the first one, 10 × 10^6^ (low dose) or 100 × 10^6^ (high dose) BM-MSCs were administered in 1.5 and 3 ml doses respectively. Subsequently, 4 ml HA (Hyalone^®^) were injected using the same via.

### Outcomes of interest

The occurrence of complications and/or adverse effects during the study was registered. In addition, the response to the intra-articular infusion of HA with or without BM-MSCs was assessed using the following procedures:

A goniometer-based evaluation of the articular range of motion at baseline i.e. before treatment administration, and 3, 6 and 12 months after treatment.

*Two scale-based methods* Visual Analog Scale (VAS) [25] and the Likert version of the Western Ontario and McMaster Universities Osteoarthritis Index (WOMAC) [26, 27], evaluated at baseline and 3, 6 and 12 months after treatment, to clinically assess pain and function. VAS ranges from 0 (maximum relief, i.e., no pain) to 10 (no relief, i.e., maximal pain). WOMAC comprises three subscores: pain, which includes 5 items; stiffness, with 2 items; and physical function, with 17 items. According to previous literature, patients were considered WOMAC responders when they reported an improvement of 20 % in at least two items together with an improvement of ten points in the overall scale [28].

Rosenberg X-ray projections at baseline and 6 and 12 months afterwards to provide a radiographic assessment of the joint space width. A custom methacrylate patient positioner was used to achieve a comparative view (Additional file 2: Figure S2).

A magnetic resonance imaging (MRI) study at baseline and 6 and 12 months after treatment. Two experienced radiologists evaluated MRI images in a blinded manner by assessing the number and location of the lesions, cartilage thickness, signal intensity, and subchondral bone alteration and volume, following the Whole-Organ Magnetic Resonance Imaging Score (WORMS) protocol, in which higher score values indicate more damage [29]. 3T Magnetom TRIO equipment (Siemens, Erlangen, Germany) was used following a protocol which included an axial T1 weighted image (WI) with slice thickness of 5 mm, coronal T1 WI (4 mm), sagittal T1 WI (4 mm), sagittal T2 FS WI (4 mm) and sagittal gradient echo 3D (DESS) (2 mm).

### Statistics

The analyses were performed according to treatment assignment, and all available data from all patients were included in the analyses, following the intention-to-treat principle. Descriptive data summaries are presented as median [interquartile range (IQR)] or percentages. Within each group, the comparison of each clinical and radiographic endpoint between the value obtained at 6 or 12 months and the baseline value, i.e. the one obtained immediately before the administration of the treatment, was performed using the Mann–Whitney U test. Changes in the same end points over time were determined calculating the differences between the measurements collected at the 6 or 12-month follow-up visit and the baseline visit. Subsequently, comparisons between treatment groups were carried out using the Kruskal–Wallis test and the Mann–Whitney U test. All tests were two-tailed. A p value of 0.05 was considered to indicate statistical significance, without adjustment for multiple testing. All analyses were performed using Stata 14 (StataCorp. 2015. Stata Statistical Software: Release 14. College Station, TX: StataCorp LP) and IBM SPSS Statistics 20 (IBM Corp. Released 2011. IBM SPSS Statistics for Windows, Version 20.0. Armonk, NY: IBM Corp).

## Results

### Demographics of patients

**Fig. 1:**
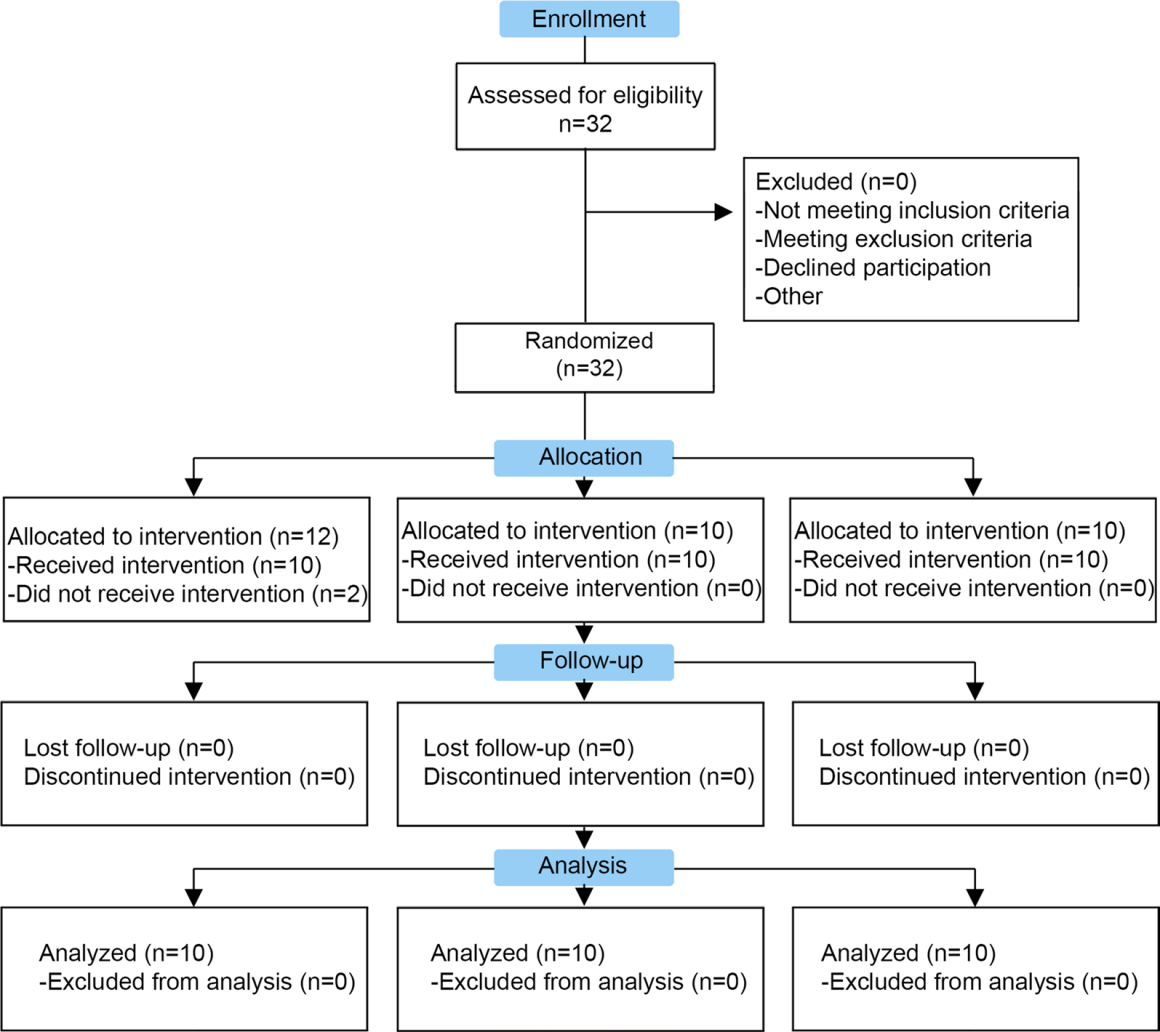
Study flow diagram. Patients were screened in the two participating centers by using the inclusion and exclusion criteria

Thirty-two patients were assessed for eligibility, and were consecutively randomized to treatment groups (Fig. 1). Two patients who had been randomly assigned to the control group withdrew consent and were excluded from the trial. All the groups showed similar baseline characteristics of age and body mass index. Patients in the three groups showed an uneven distribution according to the Kellgren–Lawrence scale but without statistical significance (p = 0.585, Table 1).

**Table 1:** Baseline characteristics of patients

	Control	BM-MSCs	
		Low-dose	High-dose
N	10	10	10
Age (years)	60.3 (55.1, 61.1)	65.9 (59.5, 70.6)	57.8 (55.0, 60.8)
Males, n (%)	7 (70)	4 (40)	8 (80)
BMI (kg/m^2^)	29.6 (26.2, 30.8)	27.1 (24.4, 31.2)	28.5 (25.8, 31.0)
Time since OA diagnosis (years)	6 (2, 8)	9 (4, 12)	10 (7, 15)
K-L 2, n (%)	4 (40)	1 (10)	3 (30)
K-L 3, n (%)	2 (20)	2 (20)	3 (30)
K-L 4, n (%)	4 (40)	7 (70)	4 (40)

Unless specified, data are presented as median [interquartile range (IQR)]. *OA* osteoarthritis, *K–L* Kellgren and Lawrence grading scale of severity of knee OA

### Safety

No serious adverse events or complications derived from the procedures or treatments were noted. There were no clinically important trends in the results of physical examination, vital signs and laboratory tests during the study. Articular pain requiring anti-inflammatory treatment during the first 24 h after infiltration was observed in 1, 3 and 6 patients in the control, low-dose BM-MSC and high-dose BM-MSC groups respectively. All patients recovered completely without sequelae and no treatment group-dependent differences were detected in the dose of required anti-inflammatory drug or in the time that passed until recovery.

### Clinical assessment of pain and function

**Fig. 2:**
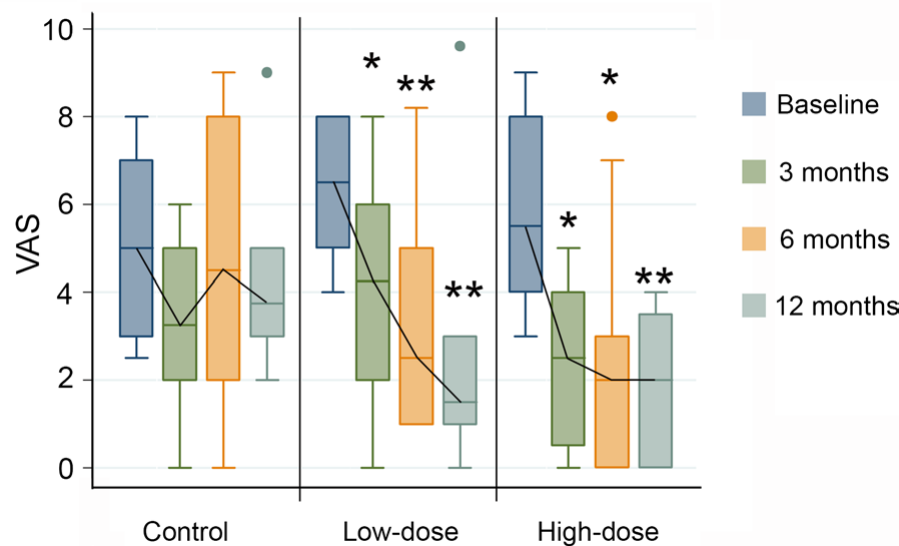
VAS scores along the study. The median values of VAS in the three groups before administration of treatments and 3, 6 and 12 months afterwards are presented. *p < 0.05; **p < 0.01 with respect to the baseline value of the same group

VAS and WOMAC clinical scores were used in order to obtain the best picture of how patients perceived their own evolution. Evaluations were performed before the administration of treatment and 3, 6 and 12 months afterwards, and the results are summarized in Fig. 2, Additional file 3: Table S1 (VAS) and Table 2 (WOMAC). The patients that were solely given HA did not show changes during follow up in their pain status according to VAS (Fig. 2; Additional file 3: Table S1). Furthermore, although they initially perceived some improvement according to the WOMAC pain and physical function subscores, this perception was not significantly sustained in the long term (Table 2). Inatraarticular delivery of BM-MSCs, specially when used at high dose, enabled patients to perceive an improvement in their perception of pain in their daily activity. On one hand, the VAS score value was significantly reduced upon treatment with low and high BM-MSC doses at all follow-up times (Fig. 2; Additional file 3: Table S1). Furthermore, treatment with 100 × 10^6^ cells was associated with a significant improvement in all WOMAC subscores at 12 months (Table 2). It is important to note that, when the overall WOMAC value at 12 months was subtracted from the baseline value in each patient, the median decrease in the score, i.e. the relief of the symptoms, was notably larger if patients had been treated with BM-MSCs [−6.5 (−19, 4), −14 (−27, 4), and −14 (−15, −8), median (IQR), for control, low-dose and high-dose BM-MSCs groups respectively]. Thus, only the patients who had been treated with BM-MSCs met criteria to be considered WOMAC responders in the long term.

**Table 2:** WOMAC score before administration of treatments and 3, 6 and 12 months afterwards

WOMAC	Time	Control	BM-MSCs	
			Low-dose	High-dose
Pain	Baseline	5.5 (5, 6)	7.5 (5, 9)	4.5 (4, 5)
	3 months	3 (1, 3)*	3.5 (3, 7)	3 (2, 5)
	6 months	2.5 (1, 5)*	3.5 (3, 7)	3.5 (2, 5)
	12 months	2 (1, 6)	3.5 (3, 5)	2.5 (2, 4)*
Stiffness	Baseline	2 (1, 3)	4 (2, 5)	2.5 (2, 4)
	3 months	2 (1, 2)	2 (0, 4)	2 (1, 2)
	6 months	0.5 (0, 2)	1.5 (1, 3)*	2 (1, 3)
	12 months	2 (1, 2)	2 (1, 2)*	2 (1, 2)*
Function	Baseline	21 (13, 24)	26.5 (23, 32)	19 (12, 25)
	3 months	9 (7, 11)*	17.5 (8, 26)	10 (7, 18)
	6 months	7.5 (2, 13)*	18 (10, 23)	14.5 (8, 17)
	12 months	9.5 (5, 23)	17 (10, 20)	11 (9, 14)*
Overall	Baseline	29 (19, 38)	37 (32, 42)	28 (16, 34)
	3 months	12 (11, 14)*	25.5 (11, 37)	13 (11, 26)*
	6 months	10 (4, 20)*	24 (13, 31)	20 (13, 23)
	12 months	13.5 (8, 33)	21.5 (15, 26)	16.5 (12, 19)**

The values of each one of the three WOMAC subscales as well as the overall WOMAC score at baseline and 3, 6 and 12 months afterwards are presented. Data are the median (IQR) of each group. Function means physical function. *p < 0.05, **p < 0.01 with respect to the baseline value of the same group

### Effect of treatments on the range of knee motion

**Fig. 3:**
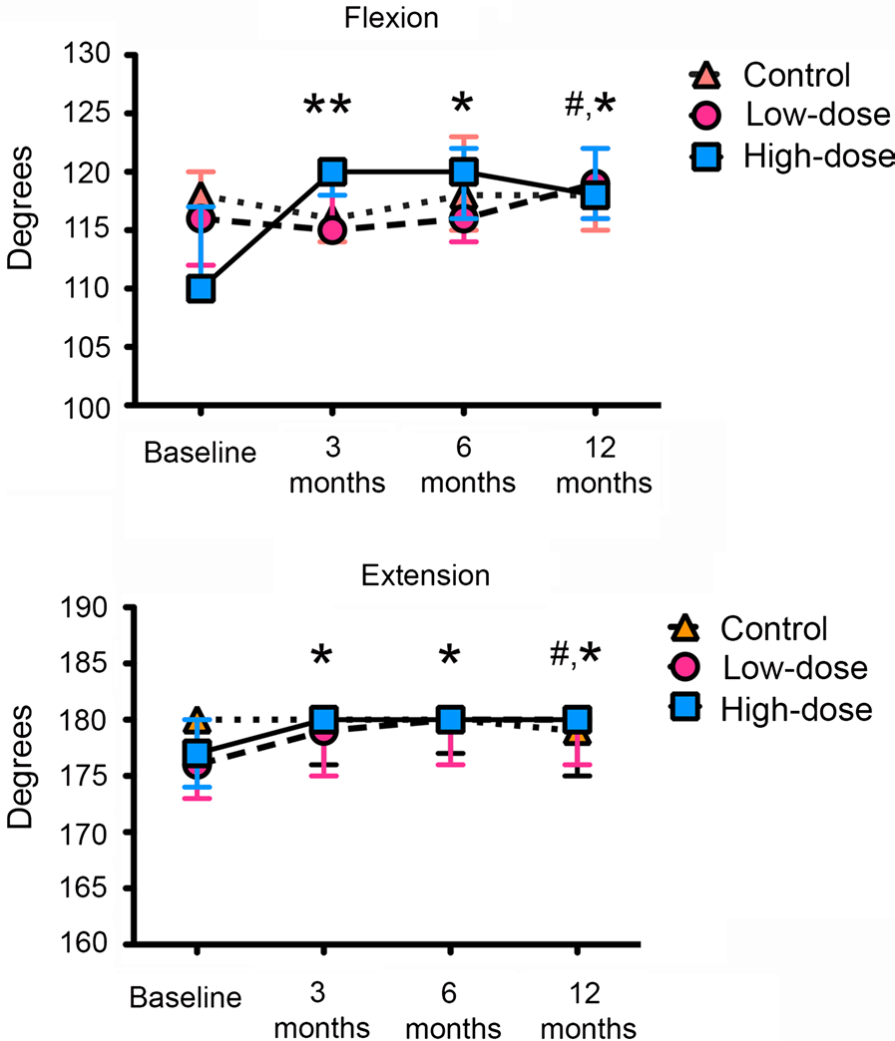
Knee range of motion along the study. The median values expressed in degrees of the goniometric measurements of the knee flexion (*top*) and extension (*bottom*) ranges of motion before administration of treatments and 3, 6 and 12 months afterwards are presented. *p < 0.05; **p < 0.01 with respect to the baseline value of the high-dose group. ^#^p < 0.05 with respect to the baseline valued of the low-dose group

The knee flexion and extension ranges of motion were significantly improved in the patients who were treated with BM-MSCs and the effect was seen earlier in patients receiving the higher doses of BM-MSC. No improvement was seen in patients receiving HA alone (Fig. 3; Additional file 4: Table S2).

### Radiological and MRI findings

The analysis of the knee joint space by X-rays during follow up showed a borderline reduction in the control group (p = 0.05 at 12 months), which was not observed in patients treated with high dose BM-MSC (Table 3; Additional file 5: Table S3). The assessment in the low dose group was not possible because the baseline value was 0. These results suggest that BM-MSC may halt the progressive loss of cartilage observed in patients with OA despite the use of HA.

**Table 3:** X-ray measurement of the evolution of the knee articular interline at 6 and 12 months after the administration of treatments

Time	Control	BM-MSCs	
		Low-dose	High-dose
6 months	−3 (−6, 0)	0 (−1, 0)	0 (−1, 1)
12 months	−4 (−18, 0)	0 (0, 3)	0 (−1, 2)

For each group of treatment, variation for knee joint space width, which was measured in mm, was calculated by subtracting, for each patient of the group, the value at 6 or 12 months from the baseline value. Data are presented as the median (IQR) of each group

Consistent with the X-Ray results, the analysis of the MRI following the WORMS protocol showed a reduction in the score value during follow up (Table 4). Patients treated with high dose BM-MSCs showed a median improvement of 4 points at 12 months, with an improvement of 22 points in 25 % of patients, while there were no signs of improvement either in the control or in the low BM-MSC group.

**Table 4:** WORMS score before administration of treatments and 6 and 12 months afterwards

Time	Control	BM-MSCs	
		Low-dose	High-dose
Baseline	79 (41, 94)	75 (64, 107)	60 (53, 84)
6 months	78 (34, 107)	70 (57, 126)	53 (51, 90)
12 months	83 (25, 95)	90 (67, 140)	53 (46, 82)
12 months evolution	−0.5 (−16, 15)	2.5 (−3, 25)	−4 (−22, 2)

The overall WORMS scores at baseline and 6 and 12 months afterwards are presented as the median (IQR) of each group. The evolution within each treatment group at 12 months is also presented, and was calculated by subtracting for each patient the values at 12 months from the corresponding baseline values. Data are the median (IQR) of each group

## Discussion

The interest in the clinical use of MSCs for the treatment of knee OA has recently grown. However, the optimal dose and source of cells, as well as the use of coadjuvants, are not yet established. In the present clinical trial we used two single doses of BM-MSCs, 10 and 100 × 10^6^ cells, coadministered with HA, and compared their effects with the single administration of HA in patients with knee OA.

We found that the use of BM-MSCs resulted in a significant relief of pain symptoms in the long term. According to the VAS scores, when BM-MSCs had been administered, an improvement was seen from the earliest evaluation and was maintained until the last one, at 12 months, at which time point the highest effect was observed. Interestingly, this pain reduction was independent of the dose of BM-MSCs administered. On the other hand, no significant changes in VAS were detected in the control group, and the value at 12 months was similar to the one registered before the administration of the treatments. Accordingly, the analysis of the information provided by WOMAC score confirmed that BM-MSCs induced relief of pain symptoms. It is interesting to note that, although treatment with HA alone was able to reduce the WOMAC score during the first 6 months, this improvement was not sustained in the long term, but when patients received BM-MSCs, a significant reduction in WOMAC score was detected at 12 months. In addition, unlike what was observed with VAS, only the high dose of BM-MSCs showed an efficient reduction in the WOMAC score. Furthermore, it is notable that only patients treated with high-dose BM-MSCs met the criteria to be considered WOMAC responders [28].

The effect of MSCs on pain improvement in knee OA is controversial and the literature provides differing accounts. One metaanalysis and a comprehensive review have been recently published on this topic. Xia et al. [17] performed a metaanalysis by managing the results of seven clinical trials, concluding that cell treatments were not able to reduce pain scores. Unfortunately, the heterogeneity in the methodology used in the different studies, with different cell production methods and dosage, precludes these authors from drawing solid conclusions. On the other hand, Rodríguez-Merchán [30] reviewed 25 articles that reported the use of intra-articular injection of MSCs in knee OA, finding that MSCs induce pain relief and functional improvement in three randomized clinical trials which, however, were not comparable to ours methodologically. One of them used bone marrow concentrate, another used peripheral blood progenitor cells, while the third one used cultured autologous BM-MSCs together with a high tibial osteotomy, which is a surgical treatment with a well-known impact on pain relief [31–33]. The number of clinical randomized trials comparing different treatment and dosage is limited. In an interesting study, Orozco et al. [24, 34] reported an improvement in pain and function with the use of a single intra-articular injection of 40 × 10^6^ cultured autologous MSCs in twelve patients. In a more comparable randomized clinical trial, using allogenic MSCs, Vega et al. reported good clinical outcomes in pain control and function when comparing the use of a single intra-articular injection of 40 × 10^6^ cultured allogenic MSCs against a single intraarticular injection of HA [23].

Osteoarthritis is not considered a classical inflammatory arthropathy due to the absence of neutrophils in the synovial fluid and the lack of systemic manifestations of inflammation [35]. However, it is frequently associated with inflammation signs and symptoms such as joint pain, swelling and stiffness, leading to significant functional impairment and disability [36]. The improvement in pain scores together with the mild effect on function and MRI scores suggests that the positive effect of BM-MSCs that we have observed may rely on their paracrine function. In support of this notion, MSC antiinflammatory properties have been correlated with pain reduction elsewhere [37–40]. In addition, the reduction in pain scores may explain the positive changes in flexion and extension. Although such changes are small, it must be noted that a limitation of only a few degrees in flexo-extension may severely compromise the daily functional activity. These improvements together with the findings in the image analyses, suggest that MSC-based therapies may be indicated in asymptomatic patients with mild OA grade, in whom the injected MSCs could be more effective through their paracrine function when a healthier cartilage is still present.

The maintenance of the knee joint space width has been related to an appropriate cartilage thickness [41]. Unlike what happened in the patients that were treated with HA only, who experienced a reduction of this space over the time of the study, the space width was preserved when BM-MSC were also administered, even though the results obtained in the patients that had received the low dose must be taken cautiously since the baseline value in 25 % of them was already zero, which precludes suitable follow-up. Nevertheless, a difference could be observed between the high dose and control groups, which did exhibit comparable baseline values. This finding is consistent with MRI observations and is in agreement with previous reports that also investigated the role of cultured MSCs or MSCs embedded in scaffolds in knee OA [23, 24, 42–44].

The required dose of MSCs to treat knee OA efficiently is a topic of active research. Recently Jo et al. [12] performed a pilot study comparing three doses of cultured adipose tissue-derived MSCs (1 × 10^6^, n = 3; 50 × 10^6^, n = 3; and 100 × 10^6^, n = 3). They found a significant reduction in the VAS score only in the high dose group at 6 months, in spite of the small number of patients included. Since results were better with the highest dose, they focused on this in a second phase of the study 100 × 10^6^ (n = 9), with promising results. Our findings also suggest that it is preferable to administer 100 × 10^6^ rather than 10 × 10^6^ cells. However, we have to bear in mind that, despite randomization, the OA degree at recruitment was more severe in the patients who received only 10 × 10^6^ cells, which may obscure our interpretation of this result.

It is accepted that OA patients have a MSC deficit that leads to a degenerative process, and the number, in vitro proliferation and differentiation potential of BM-MSCs present in the subchondral bone decreases with age and OA grade [10, 11, 45]. However, we were able to obtain a sufficient amount of BM-MSCs in osteoarthritis patients, regardless of their age or grade of disease [46–49]. We have not identified any problems during the process of production of autologous BM-MSCs, achieving the number of autologous BM-MSCs proposed, even though the mean age of patients was around 60 years.

The present study is not exempt from limitations. First, ethical issues prevented us from performing a double-blinded trial. In order to minimize this inconvenience, subjective clinical scores were contrasted with objective measures to minimize bias. In addition, two independent radiologists carried out the MRI analyses in a blinded manner. Second, the relatively short duration of the study prevented us from analyzing the efficiency of the treatments beyond 1 year after the administration of the treatments. Finally, as anticipated, the severe initial condition of a portion of patients who were going to be administered the low dose of cells may have stopped these exerting more beneficial effects.

## Conclusions

Our study shows that the single intraarticular injection of in vitro expanded autologous BM-MSCs together with HA is a safe and feasible procedure that results in a clinical and functional improvement of knee OA, especially when 100 × 10^6^ cells are administered. These results pave the way for a future phase III clinical trial.

## Additional files

10.1186/s12967-016-0998-2 Pattern of treatment administration. BM-MSCs (bottom right inset) were administered in two consecutive intraarticular injections with a 19 G needle using a lateral patellar approach. 10 × 10^6^ or 100 × 10^6^ cells were injected in 1.5 and 3 ml respectively and subsequently 60 mg hyaluronic acid were administered in 4 ml. Patients randomized to the control group received solely the second injection.
10.1186/s12967-016-0998-2 A–C, methacrylate patient positioner to permit a correct caption of Rosenberg X-ray projections. The X ray tube is placed behind the patient, at the level of the knee and at an angle of 10° with respect to the horizontal in order to evaluate the knee articular width. D, examples of the X-ray images, obtained at baseline and 6 and 12 months afterwards, of the knees of three of the recruited patients are shown. For each patient, images are comparable to each other, which makes it possible to obtain a valid and comparable value of the articular line.
10.1186/s12967-016-0998-2 VAS before administration of treatments and 3, 6 and 12 months afterwards.
10.1186/s12967-016-0998-2 Goniometric measurements of the knee flexion and extension ranges of motion before administration of treatments and 3, 6 and 12 months afterwards.
10.1186/s12967-016-0998-2 X-ray measurement of the knee articular interline before administration of treatments and 6 and 12 months afterwards.

## Abbreviations

αMEM: alpha minimum essential medium
bFGF: fibroblast growth factor
BM-MSCs: bone marrow mesenchymal stromal cells
GMP: good manufacture practices
HA: hyaluronic acid
ISCT: International Society for Cellular Therapy
K-L: Kellgren and Lawrence scale
MRI: magnetic resonance imaging
MSCs: mesenchymal stromal cells
OA: osteoarthritis
VAS: visual analogue scale
WOMAC: Western Ontario and McMaster Universities Osteoarthritis Index
WORMS: Whole-Organ Magnetic Resonance Imaging Score

## References

[1] Ishiguro N, Kojima T, Poole AR (2002). Mechanism of cartilage destruction in osteoarthritis. Nagoya J Med Sci.

[2] Mazor M, Lespessailles E, Coursier R, Daniellou R, Best TM, Toumi H (2014). Mesenchymal stem-cell potential in cartilage repair: an update. J Cell Mol Med.

[3] Simon LS (1999). Osteoarthritis. Curr Rheumatol Rep.

[4] Buckwalter JA, Saltzman C, Brown T (2004). The impact of osteoarthritis: implications for research. Clin Orthop Relat Res.

[5] Steinert AF, Ghivizzani SC, Rethwilm A, Tuan RS, Evans CH, Noth U (2007). Major biological obstacles for persistent cell-based regeneration of articular cartilage. Arthritis Res Ther.

[6] Knutsen G, Drogset JO, Engebretsen L, Grontvedt T, Isaksen V, Ludvigsen TC, Roberts S, Solheim E, Strand T, Johansen O (2007). A randomized trial comparing autologous chondrocyte implantation with microfracture. Findings at five years. J Bone Joint Surg Am.

[7] Brittberg M, Lindahl A, Nilsson A, Ohlsson C, Isaksson O, Peterson L (1994). Treatment of deep cartilage defects in the knee with autologous chondrocyte transplantation. N Engl J Med.

[8] Nejadnik H, Hui JH, Feng Choong EP, Tai BC, Lee EH (2010). Autologous bone marrow-derived mesenchymal stem cells versus autologous chondrocyte implantation: an observational cohort study. Am J Sports Med.

[9] Caplan AI (2005). Review: mesenchymal stem cells: cell-based reconstructive therapy in orthopedics. Tissue Eng.

[10] Caplan AI (2007). Adult mesenchymal stem cells for tissue engineering versus regenerative medicine. J Cell Physiol.

[11] Murphy JM, Dixon K, Beck S, Fabian D, Feldman A, Barry F (2002). Reduced chondrogenic and adipogenic activity of mesenchymal stem cells from patients with advanced osteoarthritis. Arthritis Rheum.

[12] Jo CH, Lee YG, Shin WH, Kim H, Chai JW, Jeong EC, Kim JE, Shim H, Shin JS, Shin IS, Ra JC, Oh S, Yoon KS (2014). Intra-articular injection of mesenchymal stem cells for the treatment of osteoarthritis of the knee: a proof-of-concept clinical trial. Stem Cells.

[13] Campagnoli C, Roberts IA, Kumar S, Bennett PR, Bellantuono I, Fisk NM (2001). Identification of mesenchymal stem/progenitor cells in human first-trimester fetal blood, liver, and bone marrow. Blood.

[14] Karp JM, Leng Teo GS (2009). Mesenchymal stem cell homing: the devil is in the details. Cell Stem Cell.

[15] Horie M, Choi H, Lee RH, Reger RL, Ylostalo J, Muneta T, Sekiya I, Prockop DJ (2012). Intra-articular injection of human mesenchymal stem cells (MSCs) promote rat meniscal regeneration by being activated to express Indian hedgehog that enhances expression of type II collagen. Osteoarthr Cartil.

[16] Gupta PK, Das AK, Chullikana A, Majumdar AS (2012). Mesenchymal stem cells for cartilage repair in osteoarthritis. Stem Cell Res Ther.

[17] Xia P, Wang X, Lin Q, Li X (2015). Efficacy of mesenchymal stem cells injection for the management of knee osteoarthritis: a systematic review and meta-analysis. Int Orthop.

[18] Qi Y, Feng G, Yan W (2012). Mesenchymal stem cell-based treatment for cartilage defects in osteoarthritis. Mol Biol Rep.

[19] Dominici M, Le Blanc K, Mueller I, Slaper-Cortenbach I, Marini F, Krause D, Deans R, Keating A, Prockop D, Horwitz E (2006). Minimal criteria for defining multipotent mesenchymal stromal cells. The International Society for Cellular Therapy position statement. Cytotherapy.

[20] Simmons PJ, Torok-Storb B (1991). Identification of stromal cell precursors in human bone marrow by a novel monoclonal antibody, STRO-1. Blood.

[21] Burger SR (2003). Current regulatory issues in cell and tissue therapy. Cytotherapy..

[22] Fekete N, Rojewski MT, Furst D, Kreja L, Ignatius A, Dausend J, Schrezenmeier H (2012). GMP-compliant isolation and large-scale expansion of bone marrow-derived MSC. PLoS One.

[23] Vega A, Martin-Ferrero MA, Del Canto F, Alberca M, Garcia V, Munar A, Orozco L, Soler R, Fuertes JJ, Huguet M, Sanchez A, Garcia-Sancho J (2015). Treatment of knee osteoarthritis with allogeneic bone marrow mesenchymal stem cells: a randomized controlled trial. Transplantation.

[24] Orozco L, Munar A, Soler R, Alberca M, Soler F, Huguet M, Sentis J, Sanchez A, Garcia-Sancho J (2014). Treatment of knee osteoarthritis with autologous mesenchymal stem cells: two-year follow-up results. Transplantation.

[25] Huskisson EC (1974). Measurement of pain. Lancet.

[26] Bellamy N (1995). Outcome measurement in osteoarthritis clinical trials. J Rheumatol Suppl.

[27] Escobar A, Quintana JM, Bilbao A, Azkarate J, Guenaga JI (2002). Validation of the Spanish version of the WOMAC questionnaire for patients with hip or knee osteoarthritis. Western Ontario and McMaster Universities Osteoarthritis Index. Clin Rheumatol.

[28] Escobar A, Gonzalez M, Quintana JM, Vrotsou K, Bilbao A, Herrera-Espineira C, Garcia-Perez L, Aizpuru F, Sarasqueta C (2012). Patient acceptable symptom state and OMERACT-OARSI set of responder criteria in joint replacement. Identification of cut-off values. Osteoarthr Cartil.

[29] Peterfy CG, Guermazi A, Zaim S, Tirman PF, Miaux Y, White D, Kothari M, Lu Y, Fye K, Zhao S, Genant HK (2004). Whole-organ magnetic resonance imaging score (WORMS) of the knee in osteoarthritis. Osteoarthr Cartil.

[30] Rodriguez-Merchan EC (2014). Intra-articular injections of mesenchymal stem cells for knee osteoarthritis. Am J Orthop.

[31] Wong KL, Lee KB, Tai BC, Law P, Lee EH, Hui JH (2013). Injectable cultured bone marrow-derived mesenchymal stem cells in varus knees with cartilage defects undergoing high tibial osteotomy: a prospective, randomized controlled clinical trial with 2 years follow-up. Arthroscopy.

[32] Varma HS, Dadarya B, Vidyarthi A (2010). The new avenues in the management of osteo-arthritis of knee–stem cells. J Indian Med Assoc.

[33] Saw KY, Anz A, Jee CSY, Merican S, Ng RC, Roohi SA, Ragavanaidu K (2013). Articular cartilage regeneration with autologous peripheral blood stem cells versus hyaluronic acid: a randomized controlled trial. Arthroscopy.

[34] Orozco L, Munar A, Soler R, Alberca M, Soler F, Huguet M, Sentis J, Sanchez A, Garcia-Sancho J (2013). Treatment of knee osteoarthritis with autologous mesenchymal stem cells: a pilot study. Transplantation.

[35] Goldring MB, Goldring SR (2007). Osteoarthritis. J Cell Physiol.

[36] Felson DT (2006). Clinical practice. Osteoarthritis of the knee. N Engl J Med.

[37] Uccelli A, Moretta L, Pistoia V (2008). Mesenchymal stem cells in health and disease. Nat Rev Immunol.

[38] Doorn J, Moll G, Le Blanc K, van Blitterswijk C, de Boer J (2012). Therapeutic applications of mesenchymal stromal cells: paracrine effects and potential improvements. Tissue Eng Part B Rev.

[39] Salgado AJ, Reis RL, Sousa NJ, Gimble JM (2010). Adipose tissue derived stem cells secretome: soluble factors and their roles in regenerative medicine. Curr Stem Cell Res Ther.

[40] Abumaree M, Al Jumah M, Pace RA, Kalionis B (2012). Immunosuppressive properties of mesenchymal stem cells. Stem Cell Rev.

[41] Buckland-Wright JC, Macfarlane DG, Lynch JA, Jasani MK, Bradshaw CR (1995). Joint space width measures cartilage thickness in osteoarthritis of the knee: high resolution plain film and double contrast macroradiographic investigation. Ann Rheum Dis.

[42] Centeno CJ, Busse D, Kisiday J, Keohan C, Freeman M, Karli D (2008). Increased knee cartilage volume in degenerative joint disease using percutaneously implanted, autologous mesenchymal stem cells. Pain Physician.

[43] Emadedin M, Aghdami N, Taghiyar L, Fazeli R, Moghadasali R, Jahangir S, Farjad R, Baghaban Eslaminejad M (2012). Intra-articular injection of autologous mesenchymal stem cells in six patients with knee osteoarthritis. Arch Iran Med.

[44] Kim YS, Choi YJ, Lee SW, Kwon OR, Suh DS, Heo DB, Koh YG (2015). Assessment of clinical and MRI outcomes after mesenchymal stem cell implantation in patients with knee osteoarthritis: a prospective study. Osteoarthr Cartil.

[45] Chua KH, Zaman Wan Safwani WK, Hamid AA, Shuhup SK, Mohd Haflah NH, Mohd Yahaya NH (2014). Retropatellar fat pad-derived stem cells from older osteoarthritic patients have lesser differentiation capacity and expression of stemness genes. Cytotherapy.

[46] Im GI, Jung NH, Tae SK (2006). Chondrogenic differentiation of mesenchymal stem cells isolated from patients in late adulthood: the optimal conditions of growth factors. Tissue Eng.

[47] Dudics V, Kunstar A, Kovacs J, Lakatos T, Geher P, Gomor B, Monostori E, Uher F (2009). Chondrogenic potential of mesenchymal stem cells from patients with rheumatoid arthritis and osteoarthritis: measurements in a microculture system. Cells Tissues Organs.

[48] Kafienah W, Mistry S, Dickinson SC, Sims TJ, Learmonth I, Hollander AP (2007). Three-dimensional cartilage tissue engineering using adult stem cells from osteoarthritis patients. Arthritis Rheum.

[49] Scharstuhl A, Schewe B, Benz K, Gaissmaier C, Buhring HJ, Stoop R (2007). Chondrogenic potential of human adult mesenchymal stem cells is independent of age or osteoarthritis etiology. Stem Cells.

