# Supplementary figures and images for: Intra-articular injection of two different doses of autologous bone marrow mesenchymal stem cells versus hyaluronic acid in the treatment of knee osteoarthritis: multicenter randomized controlled clinical trial (phase I/II)
Source: J Transl Med. 2016 Aug 26;14(1):246. doi: 10.1186/s12967-016-0998-2 (PMC5002157; doi:10.1186/s12967-016-0998-2)

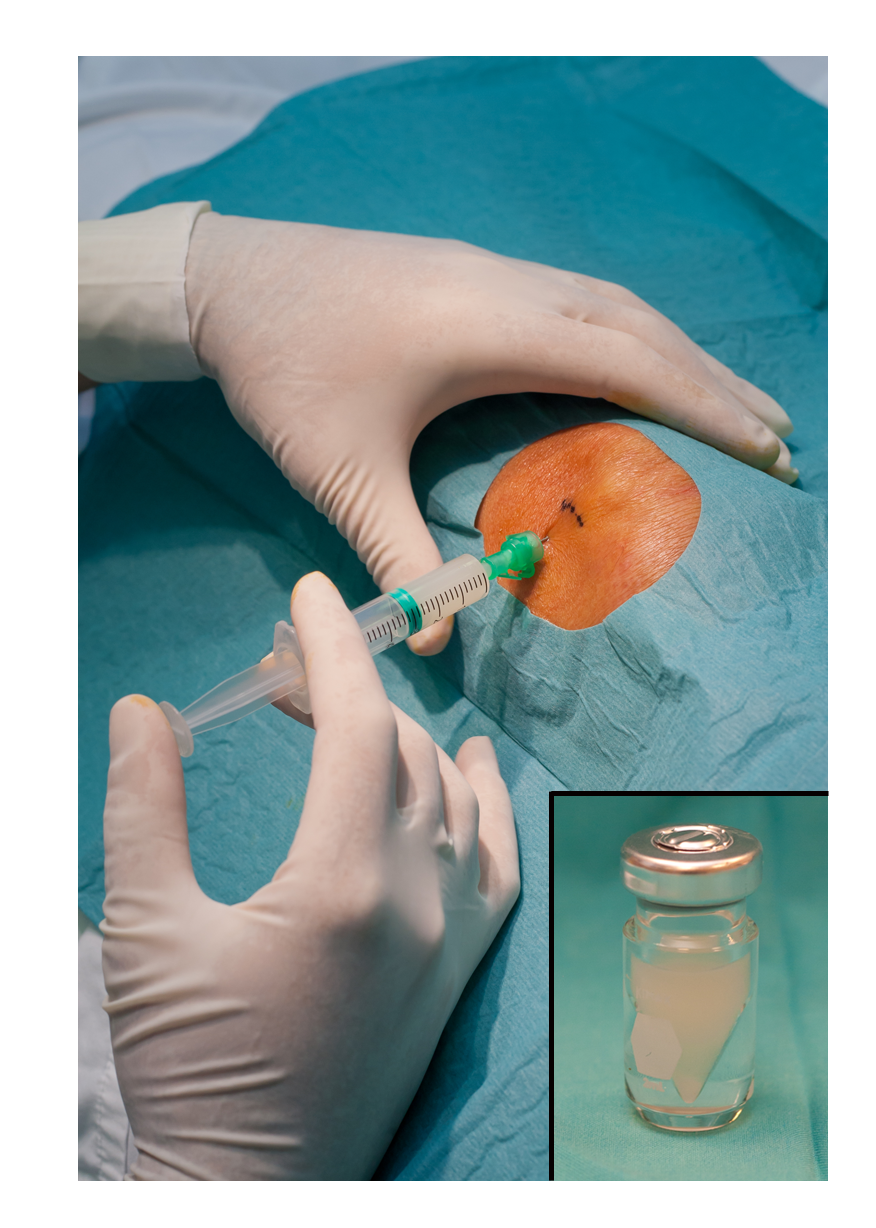

Supplement: Supplementary file 1 — 10.1186/s12967-016-0998-2 Pattern of treatment administration. BM-MSCs (bottom right inset) were administered in two consecutive intraarticular injections with a 19 G needle using a lateral patellar approach. 10 × 106 or 100 × 106 cells were injected in 1.5 and 3 ml respectively and subsequently 60 mg hyaluronic acid were administered in 4 ml. Patients randomized to the control group received solely the second injection. [file 12967_2016_998_MOESM1_ESM.tif]

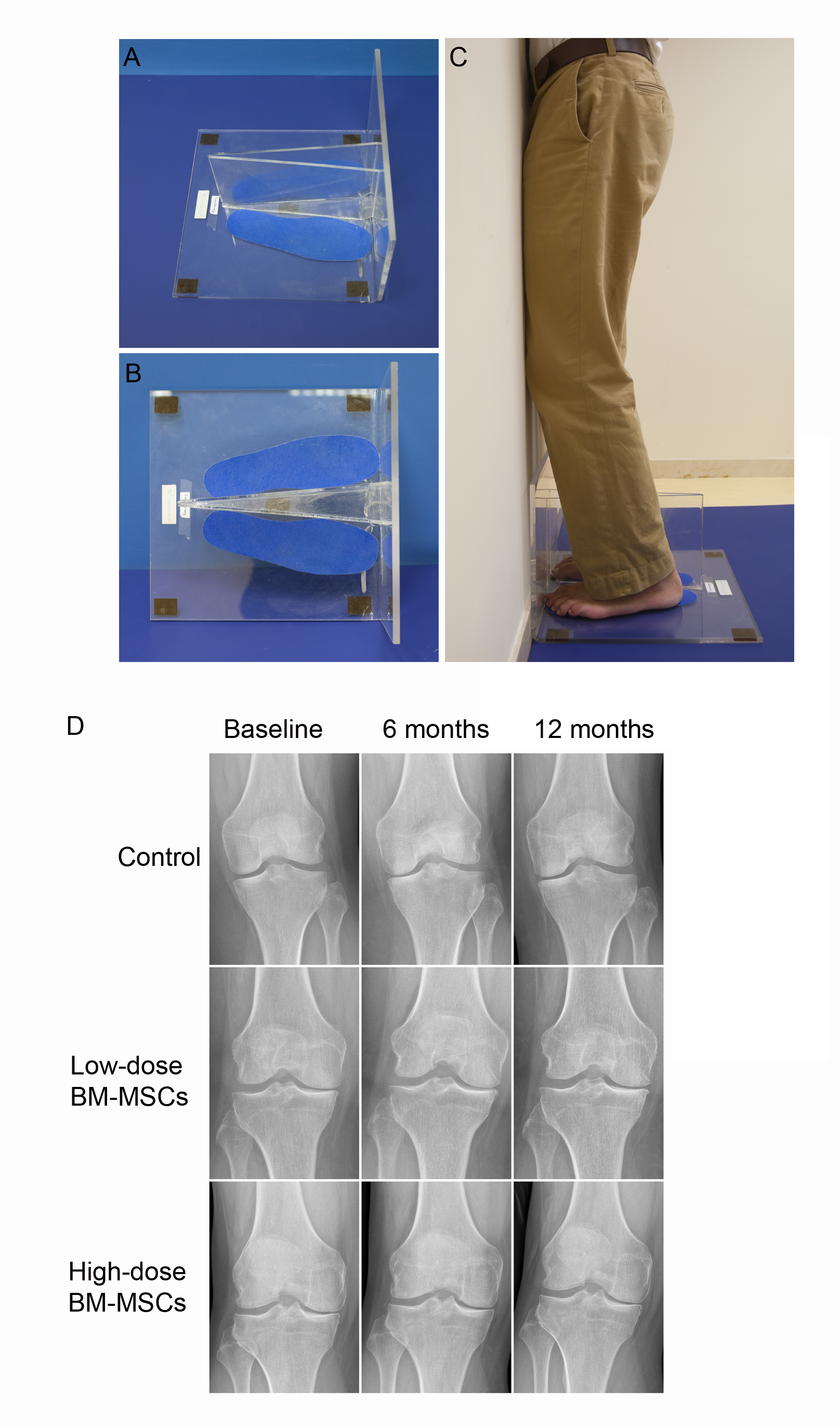

Supplement: Supplementary file 2 — 10.1186/s12967-016-0998-2 A–C, methacrylate patient positioner to permit a correct caption of Rosenberg X-ray projections. The X ray tube is placed behind the patient, at the level of the knee and at an angle of 10° with respect to the horizontal in order to evaluate the knee articular width. D, examples of the X-ray images, obtained at baseline and 6 and 12 months afterwards, of the knees of three of the recruited patients are shown. For each patient, images are comparable to each other, which makes it possible to obtain a valid and comparable value of the articular line. [file 12967_2016_998_MOESM2_ESM.tif]
