# Supplementary material for: Intra-articular injection of two different doses of autologous bone marrow mesenchymal stem cells versus hyaluronic acid in the treatment of knee osteoarthritis: multicenter randomized controlled clinical trial (phase I/II)
Source: J Transl Med. 2016 Aug 26;14(1):246. doi: 10.1186/s12967-016-0998-2 (PMC5002157; doi:10.1186/s12967-016-0998-2)
Supplement: Supplementary file 3 — 10.1186/s12967-016-0998-2 VAS before administration of treatments and 3, 6 and 12 months afterwards. [file 12967_2016_998_MOESM3_ESM.docx]

**Supplemental table 1.** VAS before administration of treatments and 3, 6 and 12 months afterwards.

| **Time** | **Control** | **BM-MSCs** | |
| --- | --- | --- | --- |
|  |  | **Low-dose** | **High-dose** |
| **Baseline** | 5 (3, 7) | 7 (5, 8) | 6 (4, 8) |
| **3 months** | 3 (2, 5) | **4 (2, 6)*** | **3 (1, 4)*** |
| **6 months** | 5 (2, 8) | **3 (1, 5)**** | **2 (0, 3)*** |
| **12 months** | 4 (3, 5) | **2 (1, 3)**** | **2 (0, 4)**** |

The values of the VAS scale at baseline and 3, 6 and 12 months afterwards are presented. Data are the median (IQR) of each group. *, p<0.05; **, p<0.01 with respect to the baseline value of the same group.
