# Supplementary material for: Intra-articular injection of two different doses of autologous bone marrow mesenchymal stem cells versus hyaluronic acid in the treatment of knee osteoarthritis: multicenter randomized controlled clinical trial (phase I/II)
Source: J Transl Med. 2016 Aug 26;14(1):246. doi: 10.1186/s12967-016-0998-2 (PMC5002157; doi:10.1186/s12967-016-0998-2)
Supplement: Supplementary file 4 — 10.1186/s12967-016-0998-2 Goniometric measurements of the knee flexion and extension ranges of motion before administration of treatments and 3, 6 and 12 months afterwards. [file 12967_2016_998_MOESM4_ESM.docx]

**Supplemental table 2**. Goniometric measurements of the knee flexion and extension ranges of motion before administration of treatments and 3, 6 and 12 months afterwards.

| **Variable** | **Time** | **Control** | | **BM-MSCs** | |
| --- | --- | --- | --- | --- | --- |
|  |  |  | **Low-dose** | | **High-dose** |
| **Flexion** | **Baseline** | 118 (114, 120) | 116 (110, 116) | | 110 (110, 117) |
|  | **3 months** | 116 (114, 120) | 115 (114, 118) | | **120 (118, 120)**** |
|  | **6 months** | 118 (115, 120) | 116 (114, 120) | | **120 (116, 122)*** |
|  | **12 months** | 118 (115, 118) | **119 (116, 122)*** | | **118 (116, 122)*** |
| **Extension** | **Baseline** | 180 (176, 180) | 176 (173, 180) | | 177 (174, 180) |
|  | **3 months** | 180 (176, 180) | 179 (175, 180) | | **180 (180, 180)*** |
|  | **6 months** | 180 (177, 180) | 180 (176, 180) | | **180 (180, 180)*** |
|  | **12 months** | 179 (175, 180) | **180 (176, 180)*** | | **180 (180, 180)*** |

The values correspond to the degrees of the knee flexion and extension ranges of motion at baseline and 3, 6 and 12 months afterwards and are presented as the median (IQR) of each group. *, p<0.05; **, p<0.01 with respect to the baseline value of the same group.
