# Supplementary material for: Intra-articular injection of two different doses of autologous bone marrow mesenchymal stem cells versus hyaluronic acid in the treatment of knee osteoarthritis: multicenter randomized controlled clinical trial (phase I/II)
Source: J Transl Med. 2016 Aug 26;14(1):246. doi: 10.1186/s12967-016-0998-2 (PMC5002157; doi:10.1186/s12967-016-0998-2)
Supplement: Supplementary file 5 — 10.1186/s12967-016-0998-2 X-ray measurement of the knee articular interline before administration of treatments and 6 and 12 months afterwards. [file 12967_2016_998_MOESM5_ESM.docx]

**Supplemental table 3**. X-ray measurement of the knee articular interline before administration of treatments and 6 and 12 months afterwards.

| **Time** | **Control** | **BM-MSC** | |
| --- | --- | --- | --- |
|  |  | **Low-dose** | **High-dose** |
| **Baseline** | 25.5 (20, 40) | 10 (0, 42) | 33.5 (13, 52) |
| **6 months** | 24 (15, 29) | 9 (0, 45) | 30 (15, 53) |
| **12 months** | 22 (0, 26) | 10 (0, 48) | 32 (15, 52) |

The values, in mm, corresponding to the knee joint space width at baseline and 6 and 12 months afterwards are presented as the median (IQR) of each group.
